# The diagnostic yield of the first episode of a periodic health evaluation: a descriptive epidemiology study

**DOI:** 10.1186/1472-6963-12-137

**Published:** 2012-05-30

**Authors:** Cindy A Kermott, Carol S Kuhle, Stephanie S Faubion, Ruth E Johnson, Donald D Hensrud, Mohammad Hassan Murad

**Affiliations:** 1 Division of Preventive Medicine, Mayo Clinic, 200 First Street SW, Rochester, MN, 55905, USA

**Keywords:** Periodic health evaluation, Annual examination, Diagnostic yield, Screening

## Abstract

**Background:**

The benefits of a periodic health evaluation remain debatable. The incremental value added by such evaluations beyond the delivery of age appropriate screening and preventive medicine recommendations is unclear.

**Methods:**

We retrospectively collected data on a cohort of consecutive patients presenting for their first episode of a comprehensive periodic health evaluation. We abstracted data on new diagnoses that were identified during this single episode of care and that were not trivial (i.e., required additional testing or intervention).

**Results:**

The cohort consisted of 491 patients. The rate of new diagnoses per this single episode of care was 0.9 diagnoses per patient. The majority of these diagnoses was not prompted by patients’ complaints (71%) and would not have been identified by screening guidelines (51%). Men (odds ratio 2.67; 95% CI, 1.76, 4.03) and those with multiple complaints at presentation (odds ratio 1.12; 95% CI, 1.05, 1.19) were more likely to receive a clinically relevant diagnosis at the conclusion of the visit. Age was not a predictor of receiving a diagnosis in this cohort.

**Conclusion:**

The first episode of a comprehensive periodic health evaluation may reveal numerous important diagnoses or risk factors that are not always identified through routine screening.

## Background

The benefits of a periodic health evaluation (PHE) were first identified in the 1920’s after individuals who underwent an evaluation to qualify for life insurance coverage were noted to have decreased mortality [1]. This evaluation has evolved into a traditional annual PHE with various components that may include obtaining a medical history, physical examination, and laboratory testing and may lead to the diagnosis of existing medical conditions and development of a plan of action. This evolution was not driven by any formal process or documented outcomes [2].

Experts have advocated for the delivery of preventive services in the context of ongoing clinical care (i.e., office visits that are not necessarily dedicated for preventive care) [3-5]. The importance of delivering preventive services has been used as a rationale to justify the PHE [6,7]. A systematic review of the literature demonstrates that a PHE is consistently associated with patient receipt of a gynecologic examination, Papanicolaou smear, cholesterol screening, and fecal occult blood testing [8]. The PHE had a beneficial effect on patient worry in one randomized controlled trial [9]. Yet, economic realities have transformed the typical general medical care visit into a shorter, problem-focused visit that may or may not contain the delivery of preventive services. Yarnall et al. estimates that to fully satisfy the United States Preventive Services Task Force (USPSTF) recommendations in a panel of 2500 patients with an age and sex distribution similar to that of the US population, a total of 1773 hours of a physician's annual time, or 7.4 hours per working day, are needed [10]. Thus, preventive medicine measures delivered in this model may become generic, untailored or inadequate due to lack of adequate provider time.

Furthermore, in addition to delivering the appropriate preventive measures, a PHE may lead to the identification of symptoms or signs that prompt testing and investigation, which is referred to as case-finding. Patients frequently use their annual exam or PHE to bring up complaints and concerns they have “saved” to be addressed on that day. The benefits of delivering preventive measures and case findings should be balanced against cost, harms associated with diagnostic interventions, and known challenges that relate to the early identification of conditions that have slow progression with minimal benefit attributed to early diagnosis and treatment.

The purpose of this study is to evaluate the diagnostic yield of the first episode of a comprehensive medical evaluation in patients seeking a preventive evaluation. The study will provide prevalence data of new, previously undiagnosed, medical conditions and risk factors that are expected to be encountered in the first episode in such a setting.

## Methods

We collected data on a sample of 500 consecutive new patients presenting for their first episode of a comprehensive PHE. For the purposes of this study, an episode is defined as an initial visit and one or more follow up visits to review results of testing. Patients were adults enrolled in an Executive Health Program designed to provide health services and evaluation of ongoing medical conditions to employer-sponsored individuals. Patients may or may not have primary care providers with whom they follow on a regular basis. The examination includes an initial 60 minute visit with a physician, electrocardiogram, and laboratory testing that consists of urinalysis, complete blood count, and basic chemistry tests (e.g., serum glucose, lipids, and liver, kidney and thyroid function tests). Physicians also provide additional testing based on patients’ symptoms, risk factors, personal and family history; and deliver immunizations and screening tests. Physicians meet for a second time with patients to discuss test results and to provide education and counseling (this is a shorter visit that averages 15–20 minutes and typically takes place the following day). The executive health cohort has a mean age of 52 years with a majority of men (68%) and a high level of education (80% with education of 4-year college or higher). The characteristics of the whole cohort, from which this sample is derived, have been described in detail elsewhere [11].

Trained nurse data abstractors recorded the diagnoses listed in the medical record after the conclusion of the medical episode (i.e., after the second or final encounter). Diagnoses were extracted from the medical notes as dictated by physicians. To determine the yield of this medical evaluation, existing diagnoses, defined as those reported by the patient or noted in available medical records either brought by the patient or obtained by the physician during the course of the evaluation, were excluded. Only new disease diagnoses detected during this evaluation were analyzed, along with all potential significant risk factors for developing a disease.

Given a lack of valid and reliable scales to grade the clinical importance of diagnoses encountered in outpatient settings, three physicians independently rated the importance of diagnoses and excluded trivial ones (e.g., refraction disorders, cerumen impaction, cherry angiomata, excessive caffeine intake, seborrheic keratosis, skin tags and skin telangiectasias, etc.). Agreement among the three raters was assessed by estimating the intraclass correlation coefficient (ICC) in a random sample of 44 diagnoses. The ICC can be used to assess agreement among multiple raters and can be interpreted similarly to Kappa (≥ 0.75 is consistent with good agreement, 0.40-0.75 is consistent with fair agreement, and ≥ 0.4 is consistent with poor agreement). In the random sample, the agreement was excellent (0.94; 95% Confidence Interval (CI), 0.91, 0.97).

Each diagnosis was classified as either patient prompted (i.e., patient reported symptoms related to the diagnosis) or non-patient prompted (i.e., identified by data gathered from the comprehensive history and examination, as well as from age and gender appropriate laboratory investigations and USPSTF screening recommendations). We estimated the odds ratio (OR) and 95% CI of having at least one diagnosis using logistic regression with three explanatory variables, sex, age, and number of concerns or complaints listed on the admission intake form filled by patients.

The study was approved by the Mayo Clinic Institutional Review Board, Rochester, Minnesota. Only patients who had consented to have their medical record data used for research purposes were included. All analyses were performed using SAS 9.2 version software.

## Results

Of the 500 eligible consecutive new patients enrolled in the program and presenting for their first episode of care, nine refused release of their record for research, leaving 491 patients in the study population (351 men and 140 women). The cohort was diagnosed with 428 new and clinically important diagnoses (mean of 0.9 diagnoses per patient); these are further categorized below. Patients’ age in this cohort averaged 47 years (range 20–77 years). Of the 428 new diagnoses, 82% (350/428) were in men and 18% (78/428) in women. The characteristics of included patients are described in Table 1.

**Table 1:** Baseline demographics of study cohort

**Variable**	**Overall**	**No new clinically important diagnoses**	**New clinically important diagnoses**	***P*Value**
	**(N = 491)**	**(N = 240)**	**(N = 251)**	
Age, years (± 1 SD)	50.7 (± 9.5)	50.7 (± 9.3)	50.7 (± 9.7)	1
Concern, n (± 1 SD)	5.7 (± 3.4)	5.2 (± 2.9)	6.2 (± 3.8)	<.001
Diagnoses, n (± 1 SD)		9.8 (± 4.0)	1.6 (± 0.9)	<.001
Gender, n (%)				<.001
Female	140 (29)	88 (37)	52 (21)	
Male	351 (71)	152 (63)	199 (79)	
Age ranges, n (%)				0.64
20-39	59 (12)	32 (13)	27 (11)	
40-64	401 (82)	194 (81)	207 (82)	
65+	31 (6)	14 (6)	17 (7)	

These diagnoses were given to 260 unique patients (53% of all patients) with a mean of 1.6 diagnoses per patient. On further breakdown, 139 patients had one diagnosis, 90 had two diagnoses, 22 had three, 6 had four, 1 had five, 1 had six, and 1 had eight. The prevalence of diagnoses is described in Table 2..

**Table 2:** Prevalence of diagnoses

**Categories**	**Prevalence, n (%)** **N = 491**	**No. Not Patient Prompted**	**USPSTF** **Recommendationsfor Screening**
**ENDOCRINE**			
Adrenal adenoma	2 (0.4)	2	NR
Diabetes mellitus Type 2	3 (0.6)	3	B*
Dyslipidemia	40 (8.2)	40	A**
Chronic fatigue	1 (0.2)	1	NR
Hyperparathyroidism	6 (1.2)	6	NR
Hyperthyroidism	2 (0.4)	2	I
Elevated lipoprotein (a)	12 (2.4)	12	NR
Metabolic syndrome	6 (1.2)	6	NR
Obesity	95 (19.4)	88	B
Class I	72	67	
Class II	13	12	
Class III	10	9	
Osteoporosis	9 (1.8)	8	B
Thyroid nodule	4 (0.8)	2	I
Hashimoto’s thyroiditis	1 (0.2)	1	I
Thyrotoxicosis	1 (0.2)	1	I
Vertebral compression fracture	2 (0.4)	2	NR
**TOTAL**	**184 (37.4)**	**174**	
**MALIGNANCY**			
Non-melanoma skin	8 (1.6)	8	I
Melanoma	7 (1.4)	4	I
Prostate cancer	4 (0.8)	4	D
Renal cell carcinoma	1 (0.2)	1	NR
Pancreatic cancer	1 (0.2)	0	D
**TOTAL**	**21 (4.2)**	**17**	
**PRE-MALIGNANT**			
Colon polyp (large, multiple, tubulovillous+)	5 (1.0)	5	A
PSA elevation	6 (1.2)	6	D
Night sweats	2 (0.4)	0	NR
**TOTAL**	**13 (2.6)**	**11**	
**CARDIOVASCULAR**			
Aortic aneurysm, thoracic	1 (0.2)	1	NR
Aortic aneurysm, abdominal	1 (0.2)	1	B
Aortic dilatation (any area)	2 (0.4)	2	NR
Moderate aortic stenosis	1 (0.2)	1	NR
Aortic valve regurgitation	2 (0.4)	2	NR
Bicuspid aortic valve	1 (0.2)	1	NR
CAD, stable	3 (0.6)	3	D
CAD, unstable	1 (0.2)	0	NR
Chest pain	4 (0.8)	3	NR
LVH	2 (0.4)	2	NR
Patent foramen ovale	1 (0.2)	1	NR
Abnormal stress test	2 (0.4)	2	D, I***
Congestive heart failure	1 (0.2)	0	NR
Peripheral artery disease	1 (0.2)	0	D
**TOTAL**	**23 (4.6)**	**19**	
**GASTROENTEROLOGY**			
Barrett’s esophagus	1 (0.2)	1	NR
Crohn’s disease	1 (0.2)	1	NR
Diverticulitis	4 (0.8)	3	NR
Diverticulosis	1 (0.2)	1	NR
Esophagitis	6 (1.2)	5	NR
Helicobacter pylori	4 (0.8)	3	NR
Hemochromatosis	1 (0.2)	1	D
Hepatitis C	1 (0.2)	1	D
Splenomegaly	2 (0.4)	2	NR
Steatohepatitis	8 (1.6)	8	NR
Mesenteric abscess	1 (0.2)	0	NR
Hematochezia	2 (0.4)	0	NR
**TOTAL**	**32 (6.4)**	**25**	
**NEUROLOGY**			
Cerebral infarction	1 (0.2)	0	NR
Lumbar stenosis	1 (0.2)	1	NR
Neuropathy	6 (1.2)	2	NR
Paresthesia	12 (2.4)	4	NR
Pituitary adenoma	1 (0.2)	1	NR
Seizure	1 (0.2)	1	NR
Syringomyelia	1 (0.2)	1	NR
Spinal syrinx	1 (0.2)	1	NR
Gliomatosis cerebri	1 (0.2)	0	NR
Transient ischemic attack	1 (0.2)	0	NR
**TOTAL**	**26 (5.2)**	**11**	
**PULMONARY**			
COPD	3 (0.6)	3	D
Obstructive sleep apnea	24 (4.9)	11	NR
Pleural effusion	1 (0.2)	1	I
Pleural thickening	1 (0.2)	1	I
Pneumonitis	2 (0.4)	2	NR
Upper airway resistance	1 (0.2)	1	NR
Asthma	1 (0.2)	0	NR
LTBI	1 (0.2)	1	NR
**TOTAL**	**34 (6.9)**	**20**	
**NEPHROLOGY**			
IGA nephropathy	1 (0.2)	1	NR
Medullary sponge kidney	1 (0.2)	1	NR
Nephrolithiasis	3 (0.6)	2	NR
Polycystic kidney disease	2 (0.4)	2	NR
Prerenal azotemia	1 (0.2)	1	NR
Renal insufficiency	3 (0.6)	3	NR
**TOTAL**	**11 (2.2)**	**10**	
**OPHTHALMOLOGY**			
Macular degeneration	3 (0.6)	3	NR
Glaucoma	4 (0.8)	4	I
Diabetic retinopathy	1 (0.2)	1	NR
**TOTAL**	**8 (1.6)**	**8**	
**PSYCHIATRIC**			
Depression	9 (1.8)	1	B
Eating disorder	1 (0.2)	1	NR
Adjustment disorder	2 (0.4)	0	NR
Anxiety	8 (1.6)	1	NR
**TOTAL**	**20 (4.0)**	**3**	
**HEALTH HABITS**			
Alcohol abuse	5 (1.0)	1	B
Nicotine dependency	43 (8.8)	2	A
**TOTAL**	**48 (9.8)**	**3**	
**OTHER**			
Hearing loss (severe/asymmetric)	5 (1.0)	3	NR
Seronegative spondyloarthropathy	1 (0.2)	0	NR
Infertility	1 (0.2)	0	NR
Otitis media	1 (0.2)	0	NR
**TOTAL**	**8 (1.6)**	**3**	
**TOTAL**	**428 (87.0)**	**304**	

Abbreviations: PSA, prostatic surface antigen; CAD, coronary artery disease; LVH, left ventricular hypertrophy; LTBI, Latent Tuberculosis Infection; COPD, chronic obstructive pulmonary disease; USPSTF, United States Preventive Services Task Force.

Recommendations for average risk patient are considered Grade A- Strongly recommended; Grade B- Recommended; Grade C- No recommendation; Grade d- Recommend against routine use; Grade I- Insufficient evidence to recommend for or against the intervention; NR- No recommendation available.

*Screen in individuals with hypertension, 2 out of 3 individuals met this criteria.

**Screen for men age ±35 and women age ±45.

***D for adults with low risk; I for adults with increased risk for coronary heart disease. ^ elevated PSA did not result in diagnosis of prostate cancer

Of 428 new diagnoses, 124 were patient prompted, while 304 (71%) were non-patient prompted and identified through PHE screening investigations (laboratory tests, USPSTF recommended screening tests (mammogram, colonoscopy etc.) and physician evaluation (e.g. identification of a finding on physical examination that led to a new diagnosis). The highest prevalence of new diagnoses was found in endocrine disorders (184) with only 10 of 174 being patient prompted. Higher rates of diagnoses which were not patient prompted were also noted in the categories of malignancy, cardiovascular and gastrointestinal diseases. Nicotine dependency was not patient-prompted in 2 of 41 individuals who were nicotine-dependent, and alcohol abuse was not patient-prompted in 1 of 4 individuals with alcohol abuse.

Of the 428 diagnoses found, 401 (94%) had no corresponding USPSTF recommendation for screening. Only 9 diagnoses were detected on the basis of USPSTF recommendations grade A or B (i.e., services that should be offered).

Logistic regression demonstrated that the predictors of being diagnosed with at least one diagnosis were male sex (OR 2.67; 95% CI, 1.76, 4.03) and the number of concerns or complaints listed on the admission intake form filled by patients (OR 1.12; 95% CI, 1.05, 1.19) but not age; see Table 3.

**Table 3:** Logistic regression model to predict presence of at least one new diagnosis

**Parameter**	**Odds Ratio**	**95% CI**		**p-value**
		**Lower**	**Upper**	
Male sex	2.7	1.8	4.0	<.0001
No of concerns at presentation	1.1	1.1	1.2	0.0002
Age	1.0	1.0	1.0	0.5706

## Discussion

We conducted a retrospective chart review to determine the diagnostic yield of the first episode of a comprehensive prevention-focused PHE. We demonstrated that even in healthy, employed patients, numerous conditions can be identified, some of which may be serious or require additional evaluation and treatment (e.g., pituitary adenoma that may be functional or aortic aneurysm that requires serial monitoring or treatment). This study provides the prevalence estimates of various conditions including malignancies, endocrinopathies, cardiovascular, renal, gastrointestinal, neurologic, and ophthalmologic diseases encountered in this setting. Men and those with multiple complaints at presentation were more likely to receive a clinically relevant diagnosis at the conclusion of the visit. Age was not a predictor in this cohort.

More than half of the clinically significant diagnoses in our study may have been missed without a comprehensive screening strategy, as these had been neither patient prompted nor discoverable by typical USPSTF screening strategies.

Nicotine dependence and alcohol abuse, when diagnosed, had only been self-identified as a problem by some, but not all affected patients. This underscores the importance of proactively addressing these habits with the patient to support healthy lifestyle change, potentially avoiding long-term health consequences. The same is true of obesity, as 90% of those with class III obesity (BMI greater than 40) similarly did not identify weight as a significant health problem.

Our results are consistent with a trial by Fletcher et al. [12] in which the authors tested an approach of active multiphasic screening and case finding examinations that consisted of history, examination, urinalysis, basic chemistry, complete blood count, chest x-ray, electrocardiogram, pulmonary function testing, and audiometry. The case finding group had significantly more diagnoses than a control group (77 new diagnoses in 36 patients or 2.14 diagnoses per participant, of which, 25 were considered important (0.69 diagnosis per participant). Follow up one year later, demonstrated that intervention or change in medical care was required for 40% of these new diagnoses. Fletcher et al. argues that their study refutes the claim that if physicians were truly providing comprehensive care in their practice, there would be no need for additional multiphasic screening [12].

In addition to increased delivery of preventive services [8], PHEs can be associated with improvements in several other patient-important outcomes. Friedman et al. [13] conducted a randomized controlled trial in which annual examinations were provided to 5,156 men and women age 35–54 (average of 6.8 exams over 16 years) and compared their outcomes to a control group. The study group had a 30% reduction in mortality due to pre-specified potentially preventable or treatable conditions (mainly colorectal cancer and hypertension). Patrick et al. [9] compared the outcomes of usual care to a preventive services benefit package that included a health risk assessment, a health promotion visit, a disease prevention visit, and a follow up visit. They demonstrated that Medicare beneficiaries randomized to the intervention group completed more advance directives, participated in more exercise, consumed less dietary fat, and reported higher satisfaction with health, less decline in self-rated health status, and fewer depressive symptoms.

The findings of this descriptive study may not be generalizable to other practice models or patient populations. It is likely that we underestimated the prevalence of multiple conditions because our population is likely to have higher socioeconomic and educational status, and to be gainfully employed with better access to health care. Thus, a higher yield of diagnoses would be anticipated in more diverse populations with fewer resources or contact with the health care system. We did not evaluate patients’ prior experiences with care they received in primary care settings elsewhere. Such care may dictate the frequency of more detailed evaluations like the one we presented in this cohort. In a previous study, a 6889 patient sample from the same cohort [11], we demonstrated fair, although variable, adherence to preventive recommendations (ranging 62%-91%).

Further, it is unclear whether the early diagnosis of many conditions identified in this cohort is cost effective or associated with improved survival and quality of life. It is also important to recognize that the evidence supporting benefits of PHEs is hampered by the heterogeneity of the interventions (i.e., the components and variability of the health evaluations) and with the mixed results reported in the areas of costs, disability prevention, and hospitalization [8]. The results of this report should not be interpreted as evidence against the USPSTF recommendations nor does it suggest more aggressive screening strategies. Rather, these findings support a more comprehensive evaluation. The PHE is a valuable service which facilitates implementing USPSTF recommendations and increases the diagnostic yield of other underlying conditions. This study is not comparative. Therefore, we do not claim superior diagnostic ability over alternative strategies. This study does not evaluate the relative efficacy of the components of the comprehensive evaluation as these can vary according to the unique disease entity. This report also draws attention to the lack of reliable scales, rating systems, and taxonomy that aids in categorizing the importance of diagnoses encountered in outpatient settings. In the medical literature various descriptions of diagnoses exist, such as “patient-important,” “clinically-important,” “requiring medical attention,” “requiring follow up,” and many others. We believe that the establishment of a scale or rating system will inform the design of outpatient practices and the development of clinical practice guidelines.

In summary, this study demonstrates that a comprehensive medical evaluation identifies numerous clinically important conditions that may affect the way patients feel, live and survive, that would not have been diagnosed otherwise. While our findings may not be compelling for policymakers to recommend such an intervention for all individuals, those with values and preferences consistent with the desire for early detection and intervention will likely opt to have a comprehensive PHE. Patients who schedule periodic, often annual, evaluations with their health care provider or present to executive health programs are likely self-selected for such inherent values and preferences. Therefore, providing these individuals with the option of a periodic comprehensive examination would be consistent with their values and with the second principle of evidence-based medicine (Evidence alone is never sufficient to make a clinical decision. Patients’ values and preferences should always be considered) [14]. Additionally, when patient fears and anxieties are adequately addressed, unnecessary testing and excessive utilization of the health care system may be avoided. Data from this study may help with the planning and design of outpatient practices, in addition to shaping both patient and provider expectations of these types of medical encounters.

## Conclusions

The first episode of a comprehensive PHE may reveal numerous important diagnoses or risk factors that are not always identified through routine screening. The PHE that includes a comprehensive medical history and physical examination remains a valuable tool in outpatient practice.

## Abbreviations

PHE, Periodic health evaluation; USPSTF, United States preventive services task force.

## Competing interests

The authors declare that they have no financial competing interests. As authors and physicians in clinical practice, our only competing interest is that we provide medical care in the PHE model.

## Authors’ contributions

CAK, submitting author, devised the initial research proposal concept and design, further directed the data abstraction process, data analysis, interpretation, and guided the team of physician researchers for this article covering all sections. CSK, drafted our Results and Table section. SSF and REJ drafted the Discussion and Conclusion sections. DDH drafted the Introduction and provided the impetus guiding this publication. MHM provided the mentorship for our research team. All authors read and approved the final manuscript.

## Pre-publication history

The pre-publication history for this paper can be accessed here:

http://www.biomedcentral.com/1472-6963/12/137/prepub
